# *QuickStats*: Age-Adjusted Suicide* Rates,^†^ by Urbanization Level^§^ and Sex — National Vital Statistics System, 2020

**DOI:** 10.15585/mmwr.mm7136a4

**Published:** 2022-09-09

**Figure:**
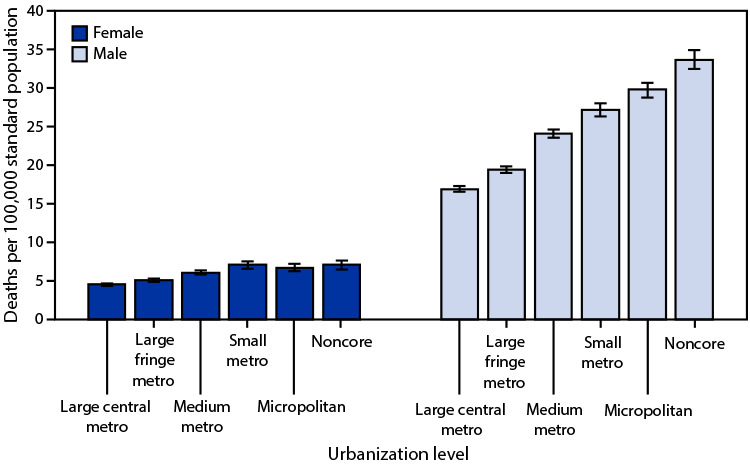
In 2020, age-adjusted suicide rates among females increased as the level of urbanization declined, from 4.6 per 100,000 population in large central metropolitan areas to 7.1 in small metropolitan areas, but were similar for small metropolitan, micropolitan, and noncore areas. Rates among males were lowest in large central areas (16.9) and increased as the level of urbanization declined to 33.7 in noncore areas. Males had higher death rates than females for each corresponding urbanization level.

**Source:** National Vital Statistics System, Mortality Data, 2020. https://www.cdc.gov/nchs/nvss/deaths.htm

For more information on this topic, CDC recommends the following link: https://www.cdc.gov/suicide

